# Downregulation of DNA repair proteins and increased DNA damage in hypoxic colon cancer cells is a therapeutically exploitable vulnerability

**DOI:** 10.18632/oncotarget.21145

**Published:** 2017-09-21

**Authors:** Jennifer M. J. Jongen, Lizet M. van der Waals, Kari Trumpi, Jamila Laoukili, Niek A. Peters, Susanne J. Schenning-van Schelven, Klaas M. Govaert, Inne H. M. Borel Rinkes, Onno Kranenburg

**Affiliations:** ^1^ UMC Utrecht, Cancer Center, 3584CX Utrecht, The Netherlands; ^2^ UMC Utrecht Division of Biomedical Genetics, 3584CX Utrecht, The Netherlands

**Keywords:** DNA repair, colon cancer, DNA damage, Tirapazamine, cancer stem cells

## Abstract

Surgical removal of colorectal cancer (CRC) liver metastases generates areas of tissue hypoxia. Hypoxia imposes a stem-like phenotype on residual tumor cells and promotes tumor recurrence. Moreover, in primary CRC, gene expression signatures reflecting hypoxia and a stem-like phenotype are highly expressed in the aggressive Consensus Molecular Subtype 4 (CMS4). Therapeutic strategies eliminating hypoxic stem-like cells may limit recurrence following resection of primary tumors or metastases.

Here we show that expression of DNA repair genes is strongly suppressed in CMS4 and inversely correlated with hypoxia-inducible factor-1 alpha (HIF1α) and HIF-2α co-expression signatures. Tumors with high expression of HIF signatures and low expression of repair proteins showed the worst survival. In human tumors, expression of the repair proteins RAD51, KU70 and RIF1 was strongly suppressed in hypoxic peri-necrotic tumor areas. Experimentally induced hypoxia in patient derived colonospheres *in vitro* or *in vivo* (through vascular clamping) was sufficient to downregulate repair protein expression and caused DNA damage. Hypoxia-induced DNA damage was prevented by expressing the hydroperoxide-scavenging enzyme glutathione peroxidase-2 (GPx2), indicating that reactive oxygen species mediate hypoxia-induced DNA damage. Finally, the hypoxia-activated prodrug Tirapazamine greatly augmented DNA damage and reduced the fraction of stem-like (Aldefluor^bright^) tumor cells *in vitro*, and *in vivo* following vascular clamping.

We conclude that decreased expression of DNA repair proteins and increased DNA damage in hypoxic tumor areas may be therapeutically exploited with hypoxia-activated prodrugs, and that such drugs reduce the fraction of Aldefluor^bright^ (stem-like) tumor cells.

## INTRODUCTION

Colorectal cancer (CRC) is the third most common cancer worldwide and a major cause of cancer-related mortality [1]. The prognosis of patients with CRC is mostly determined by the presence of distant metastases. After distant spread – predominantly to the liver – 5-year survival drops to ∼20% and the only curative treatment option is radical resection of the primary tumor and metastases. However, recurrence after liver surgery is seen in more than half of the patients [2].

Currently, treatment strategies that can limit disease recurrence following primary tumor resection or partial liver resection are not sufficiently effective. The development of such strategies should be based on an understanding of the pathways that drive metastasis and recurrence. RNA-based tumor classification has recently identified 4 distinct Consensus Molecular Subtypes (CMS1-4). CMS1 mostly consists of tumors with microsatellite instability (MSI), caused by a deficient mismatch repair system. CMS2 is the ‘canonical’ epithelial subtype with activation WNT and MYC signaling pathways. CMS3 is characterized by high expression of genes regulating metabolic pathways and is enriched in tumors with activating mutations in the *KRAS* oncogene. CMS4 is characterized by atypical expression of genes reflecting a mesenchymal and a stem cell-like phenotype and has the highest propensity to form metastases [3]. In addition, we have recently shown that mesenchymal-type primary colon tumors express high levels of hypoxia-related genes [4], which is in line with the observation that CMS4 is characterized by expression of angiogenesis-stimulating genes [3]. Hypoxia is also a driving force behind tumor recurrence following liver surgery: hypoxic tissue areas in the remnant liver form a niche for stem-like tumor cells that can subsequently drive recurrence [5, 6]. In general, hypoxia is associated with more aggressive tumor phenotypes across different types of cancer (clear cell renal carcinoma, non-small cell lung carcinoma, neuroblastoma) [7].

We hypothesized that hypoxia-targeting strategies may have value in limiting disease recurrence. Insight into the mechanisms that underlie hypoxia-stimulated tumor growth and/or the identification of vulnerabilities in hypoxic cancer cells is key to the development of such strategies. One of the consequences of hypoxia in multiple cancer types, including colon cancer, is an increased proportion of cancer stem cells (CSCs). CSCs have a high regenerative and tumorigenic potential and are generally intrinsically resistant to chemotherapy [8–13], or through indirect mechanisms [14]. Although generic CSC biomarkers are lacking and the term is used without broad consensus on the exact definition, CSCs can be operationally defined as those cells with clone- and tumor-initiating capacity. According to this pragmatic definition, aldehyde dehydrogenase (ALDH1A1) expression and activity, as measured by the Aldefluor assay, are good markers for colon CSCs [14–16].

Interestingly, hypoxia suppresses DNA repair pathways [17–21] which contributes to genomic instability [18, 21, 22]. However, impaired DNA repair capacity could also lead to an increased vulnerability to DNA-damaging agents. Hypoxia-activated prodrugs (HAPs) such as the topoisomerase-II inhibitor Tirapazamine (TPZ) can be used to target hypoxic tumor tissue [23]. Here, we have assessed the effect of hypoxia on DNA damage and DNA repair pathways in human colon cancer cells by using three-dimensional patient-derived cell cultures. We show that increased DNA damage in hypoxia is correlated with reduced expression of various DNA repair proteins, preceding tumor cell apoptosis**.** Targeting hypoxic cancer cells with TPZ further reduced DNA repair protein expression and reduced the fraction of Aldefluor^bright^ cells. Reduced repair capacity and increased DNA damage in a subset of human CRC and in post-treatment tumor tissue may provide an opportunity for therapeutic intervention with hypoxia-activated prodrugs.

## RESULTS

### Hypoxia and DNA repair in CMS4 colorectal tumors

**Figure 1:**
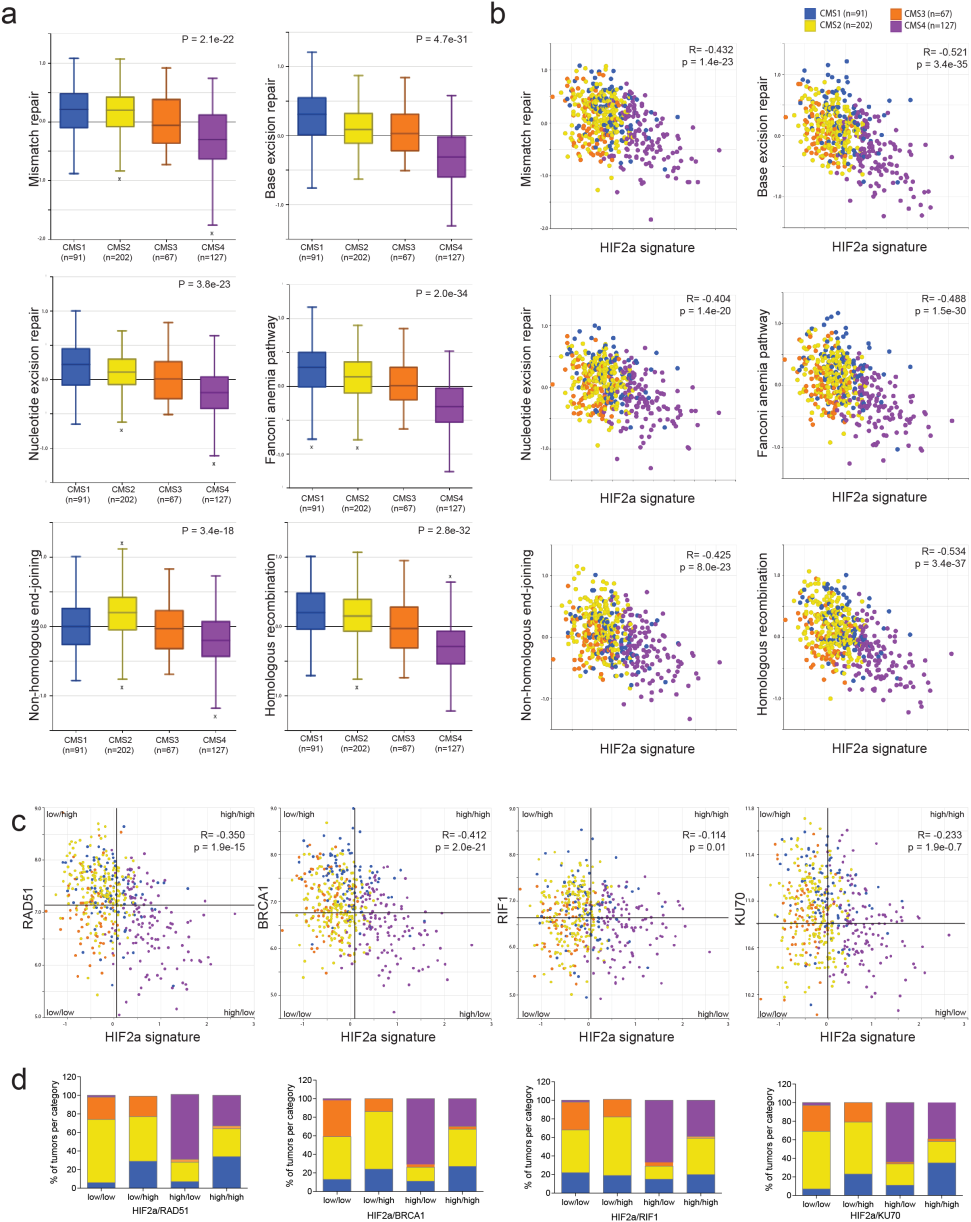
A hypoxia-inducible factor (HIF) signature is inversely correlated with expression of DNA repair genes Expression values of all genes comprising specific DNA repair pathways (www.KEGG.jp) were condensed into a single ‘meta-gene’ expression value by using the R2 platform (http://r2.amc.nl). **(a)** The box-and-whisker plots show the meta-gene expression values of 6 DNA repair pathways in relation to the 4 CMS groups (CMS1-4)[3] in the CIT dataset with CMS annotation (n=487; GSE39582). **(b)** The scatterplots show the association between the expression values of the 6 DNA repair pathway meta-genes and the expression of the HIF2α signature [4] in relation to the CMS. Blue: CMS1; yellow: CMS2; orange: CMS3; purple: CMS4. **(c)** Median expression values of 4 DNA repair genes and the HIF2α signature were used to generate quadrants (tumor subgroups). In all cases the HIF2α-high/DNA repair gene-low quadrants turn out to be mostly CMS4. **(d)** Bar graphs showing the tumor subtype distribution per quadrant.

We have previously shown that expression of a gene signature comprising the genes most significantly co-expressed with hypoxia-inducible factor 2 (HIF2α) was strongly enriched in aggressive mesenchymal-type tumors [4], now commonly referred to as CMS4. In addition, tumor hypoxia has previously been related to reduced DNA repair activity [17–19, 22]. Therefore, we studied expression of gene sets involved in specific DNA repair pathways (KEGG pathways; www.genome.jp/kegg/) and the HIF2α signature in relation to the CMSs. Strikingly, all DNA repair pathways were down-regulated in CMS4 tumors (Figure 1a) and were negatively correlated with the HIF2α signature (Figure 1b).

Next we compared HIF1α and HIF2α signatures and found highly significant co-expression in a large cohort of human CRC (Supplementary Figure 1a). Mesenchymal tumors (i.e. CMS4) expressed the highest levels of both signatures.

Many chemotherapeutic drugs cause double strand breaks, which are repaired through homologous recombination (HR) and/or non-homologous end-joining (NHEJ). We next selected single genes in these pathways, based on the magnitude of correlation (Supplementary Table 1), their use in the literature and the commercial availability of antibodies for further analyses. We found that CRC tumors with high expression of the HIF2α signature, generally had low expression of HR pathway genes (RAD51, BRCA1), and NHEJ pathway genes (RIF1, KU70) (Figure 1c and 1d). Furthermore, expression of the HIF1α signature was significantly higher in all HIF2α-HIGH/repair protein-LOW tumor subgroups (Supplementary Figure 1b). Moreover, tumor classification based on the median expression of any of these DNA repair genes and the HIF2α signature revealed that the HIF2α-HIGH/DNA-repair-LOW tumors were strongly enriched in CMS4 (Figure 1c and 1d, Supplementary Figure 1). Taken together, these data show that tumors with high expression of HIF1α and HIF2α signatures are mostly CMS4 tumors and are characterized by low expression of DNA repair genes.

### Tumors with high expression of the HIF2 signature and low expression of DNA repair genes have a poor prognosis

**Figure 2:**
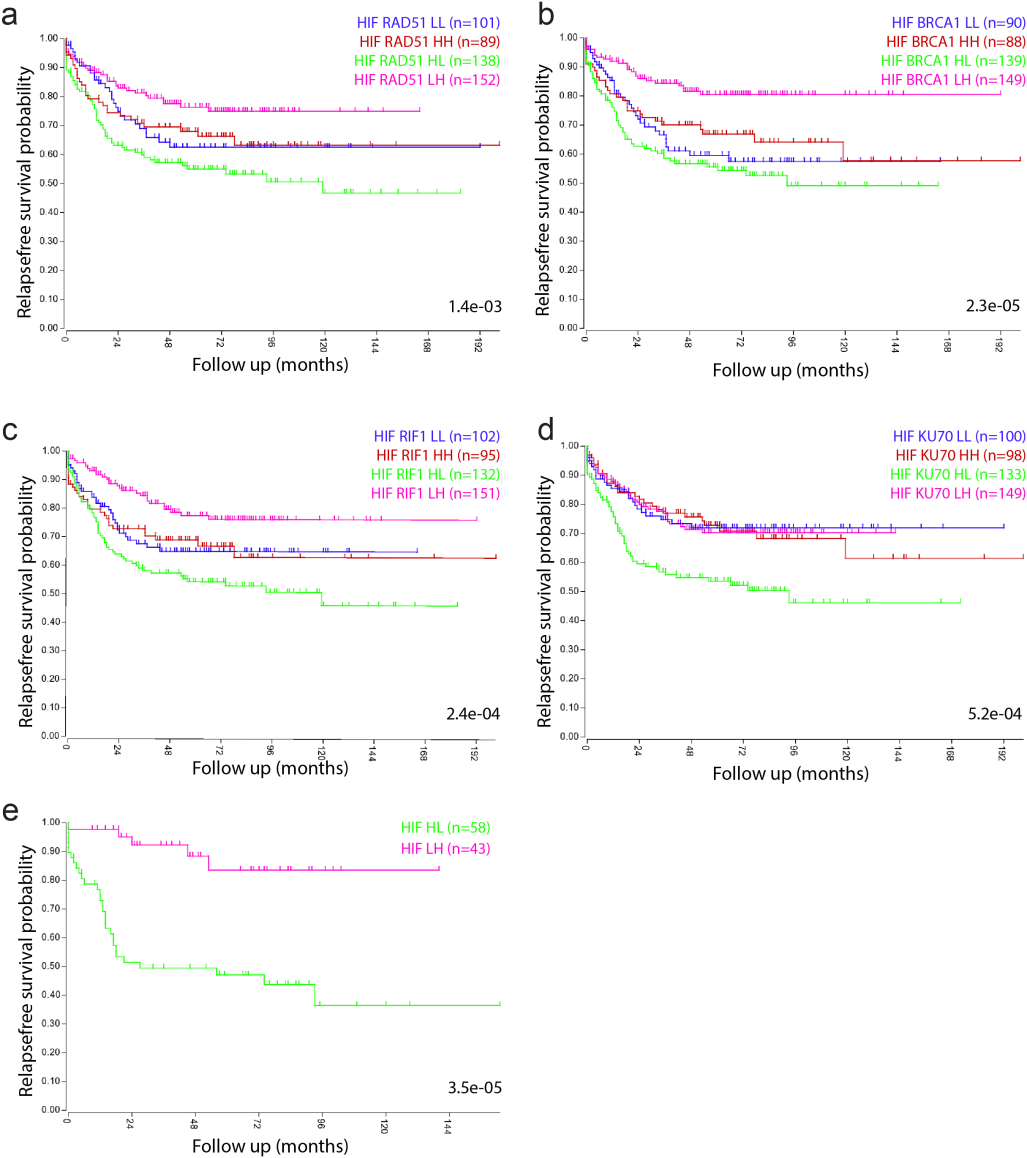
Tumors with a high hypoxia signature and low expression of DNA repair genes have a poor prognosis Kaplan Meier curves show the survival differences between the subgroups identified in Figure 1c, based on median expression values of the HIF2a signature and the indicated repair proteins. **(a)** HIF2α-BRCA1. **(b)** HIF2α-RAD51. **(c)** HIF2α-RIF1. **(d)** HIF2α-Ku70. **(e)** Tumors belonging to all 4 HIF2α-high/repair-low and to all 4 HIF2α-low/repair-high quadrants in a-d were identified by GeneVenn (www.genevenn.sourceforge.net) and analyzed separately. Tumors with high expression of the HIF2α signature and low levels of all 4 repair proteins had a very poor prognosis (green), when compared to tumors with low expression of the HIF2α signature and high expression of all 4 repair proteins (magenta).

Next, we analyzed potential differences in metastatic capacity between the different tumor subgroups identified in Figure 1. We found that of all quadrants tested survival was worst in all HIF2α-HIGH/repair-protein-LOW tumor subgroups (KM curves, green lines, Figure 2a-2d), and best in all HIF2α-LOW/repair-protein-HIGH tumor subgroups (KM curves, magenta lines, Figure 2a-2d). Next we generated tumor subgroups in which all 4 repair proteins were low and the HIF2α signature high. This revealed a remarkable difference in short and long-term survival between both subgroups: 94% and 85% of the patients with HIF-LOW-Repair-HIGH tumors remained disease-free for more than 2 and 10 years respectively, versus only 50% and 35% in the HIF-HIGH-Repair-LOW group (Figure 2e).

Similar survival differences were found when cutoff points were determined by the Kaplan-scan method, as an alternative to taking the median (Supplementary Figure 2).

### Hypoxic tumor tissue is characterized by DNA damage and low expression of RAD51 and RIF1

**Figure 3:**
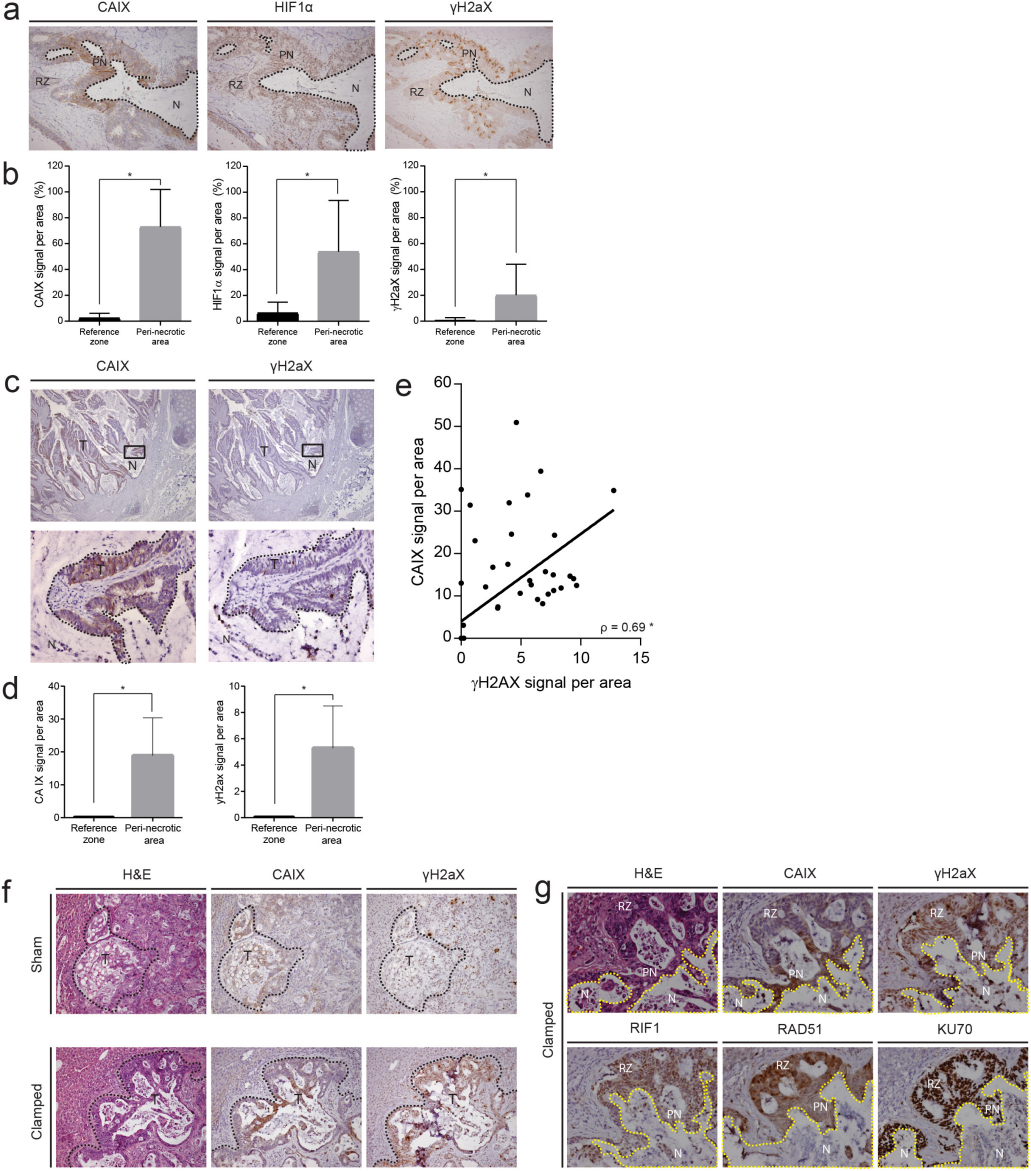
Hypoxic areas in primary tumors and liver metastases are characterized by DNA damage and low expression of the DNA repair proteins RAD51 and RIF1 **(a)** Immunohistochemistry (IHC) for CAIX, HIF1α and γH2AX in human primary colorectal tumours. Robust expression of all three markers was observed surrounding necrotic lesions. **(b)** Quantification of CAIX, HIF1α and γH2AX expression in peri-necrotic areas and reference zones in primary human colon tumors by IHC (n=20). **(c)** Analysis of CAIX and γH2AX expression in human liver metastases by IHC. A 20x magnification of the area depicted in the square in lower panel. **(d)** Quantification of CAIX and γH2AX staining (IHC) in peri-necrotic and reference tissue in liver metastases (n=30). **(e)** Scatterplot showing the correlation between the expression of (randomly chosen microscopic field areas of) CAIX and γH2AX, assessed with the Spearman test (rho) (n=60). **(f)** Patient-derived colonospheres were injected into the liver parenchyma of immune-deficient mice. Following tumor initiation, the tumor-bearing liver lobes were subjected to a vascular clamping or sham protocol [24] to induce hypoxia. After 24 hours, the livers were excised and expression of CAIX and γH2AX was examined by IHC. **(g)** IHC on peri-necrotic areas of clamped livers (experiment as in (f)) for CAIX, γH2AX, RIF1, RAD51, and KU70. * p<0.001, N=necrosis, T=tumor, PN=peri-necrotic, RZ=Reference Zone.

The data so far show an inverse correlation between HIF signatures and expression of DNA repair genes. This indicates that hypoxic tumor areas may have low repair capacity and therefore are prone to DNA damage. To study this further, we stained tissue sections of human colon tumors for the hypoxia markers CAIX and HIF1α and for the DNA damage marker phosphorylated histone 2AX (γH2AX). Peri-necrotic areas in human primary colorectal tumors and liver metastases showed strong HIF1α and CAIX staining, reflecting the hypoxic nature of this tissue. γH2AX was also highly expressed in peri-necrotic tissue, indicating increased DNA damage in these areas (Figure 3a-3e). To assess a potential causal relationship between surgery-induced hypoxia and DNA damage, we experimentally induced hypoxia in mice with pre-established liver metastases through vascular clamping as described previously [24]. Clamping caused an increase of necrotic tumor tissue, as we have shown before [5, 25, 26]. As expected, the peri-necrotic areas were characterized by strong CAIX staining. Again, γH2AX staining was also high in peri-necrotic hypoxic tumor tissue (Figure 3f). Within the clamped liver, we saw that peri-necrotic (hypoxic) areas were accompanied by a clear downregulation of the DNA repair proteins RAD51 and RIF1, while expression of KU70 was similar in hypoxic and normoxic areas (Figure 3g). These results show that both spontaneous and surgery-induced intra-tumor hypoxia suppresses expression of DNA repair proteins, which may contribute to increased DNA damage.

### Hypoxia induces DNA damage and suppresses expression of DNA repair proteins in patient-derived colonospheres

**Figure 4:**
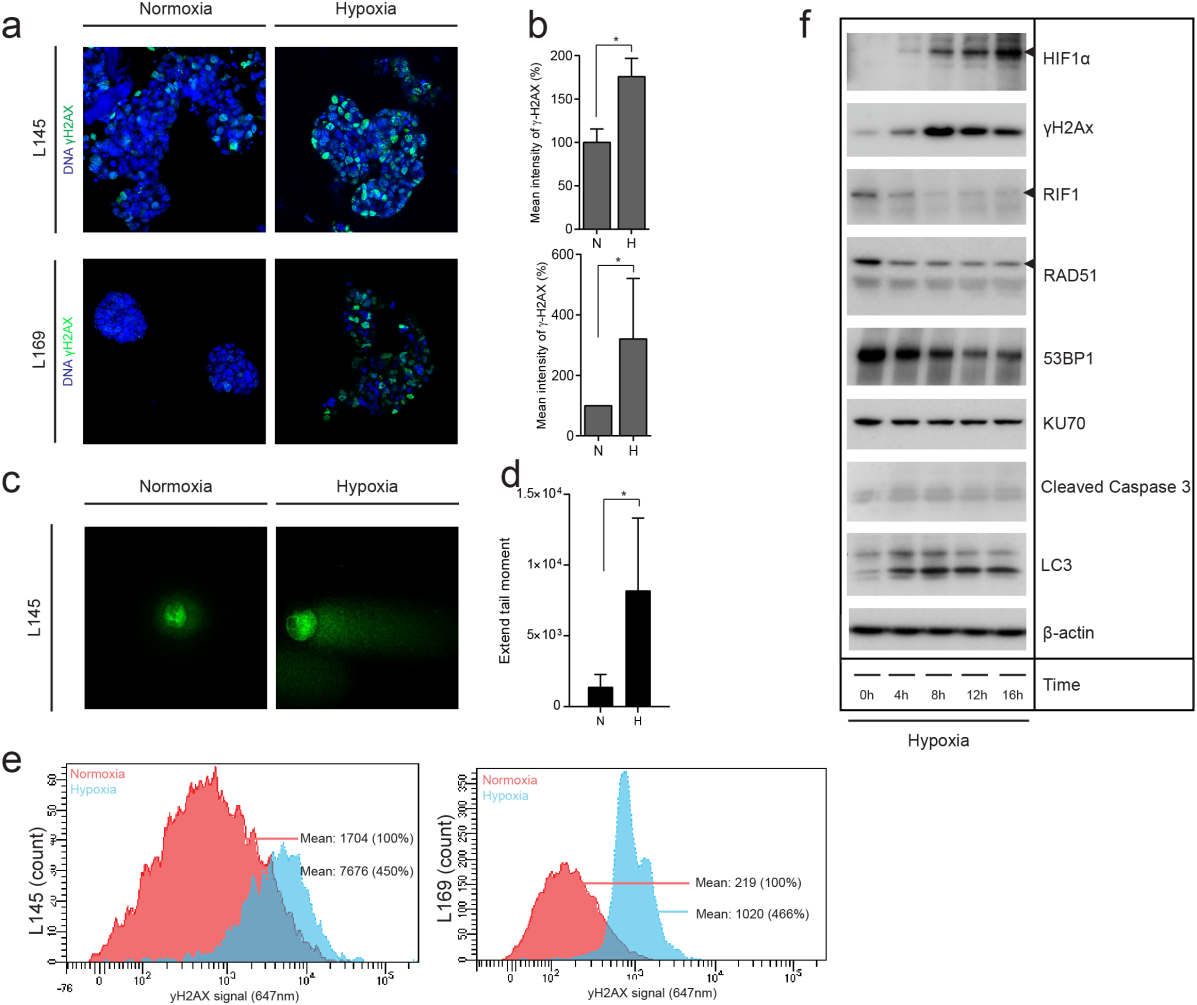
Hypoxia causes DNA damage in patient-derived colonosphere cultures *in vitro* **(a)** Two independent patient-derived colonospheres were cultured in normoxia (21%) or hypoxia (0.1%) for 24 hours. Immunofluorescence was then used to asses γH2AX (green) in the nuclei DAPI (blue). **(b)** Bar graphs showing the quantification of γH2AX observed in (a) (n=3), shown as percentage of the mean γH2AX intensity at normoxia. **(c)** Representative confocal pictures of the comet assay of colonospheres cultured as in (a). **(d)** Bar graphs showing the quantification of DNA damage observed with the comet assay (Extend tail moment) (n=73). **(e)** Colonospheres were cultured as in (a) and γH2AX was assessed by FACS. The plots show normoxic γH2AX levels in red and hypoxic γH2AX levels in blue in two independent cell lines. **(f)** Human colonospheres were exposed to hypoxia for the indicated periods of time. Cell lysates were then analyzed by Western blotting for the indicated markers (cropped).

The correlation between hypoxia and DNA damage observed in human cancer and *in vivo* experiments could be due to a direct effect of hypoxia on the tumor cells. To test this, we exposed 2 independent patient-derived colonospheres to hypoxia for 24 hours and analyzed DNA damage by γH2AX immunofluorescence (Figure 4a and 4b), Comet assays (Figure 4c and 4d) and γH2AX FACS analysis (Figure 4e). All analyses showed that hypoxia causes a marked increase in γH2AX in both colonosphere cultures. Apoptotic cells can acquire a strong secondary γH2AX signal resulting from the activation of caspase-activated DNAse (CAD) and the resulting DNA cleavage. To test whether hypoxia-induced DNA damage in human colonospheres was secondary to caspase activation we analyzed HIF stabilization, γH2AX accumulation, DNA repair protein expression, autophagy and caspase cleavage over time. Hypoxia induced a rapid and strong induction of HIF1α which was accompanied by an equally rapid accumulation of γH2AX (Figure 3f). Likewise, hypoxia induces HIF2α stabilization in these cells (Supplementary Figure 3). The induction of DNA damage was accompanied by simultaneous suppression of the DNA repair proteins RAD51, RIF1 and 53BP1 and, to a lesser extent, KU70. Importantly, during the 16-hour time frame of the experiment, hypoxia did increase autophagy (as measured by LC3 cleavage) but did not affect caspase activation (Figure 4f). The latter result shows that hypoxia-induced suppression of DNA repair proteins and increased DNA damage are not secondary to caspase activation and apoptosis.

### Overexpression of glutathione peroxidase-2 inhibits hypoxia-induced DNA damage and apoptosis

**Figure 5:**
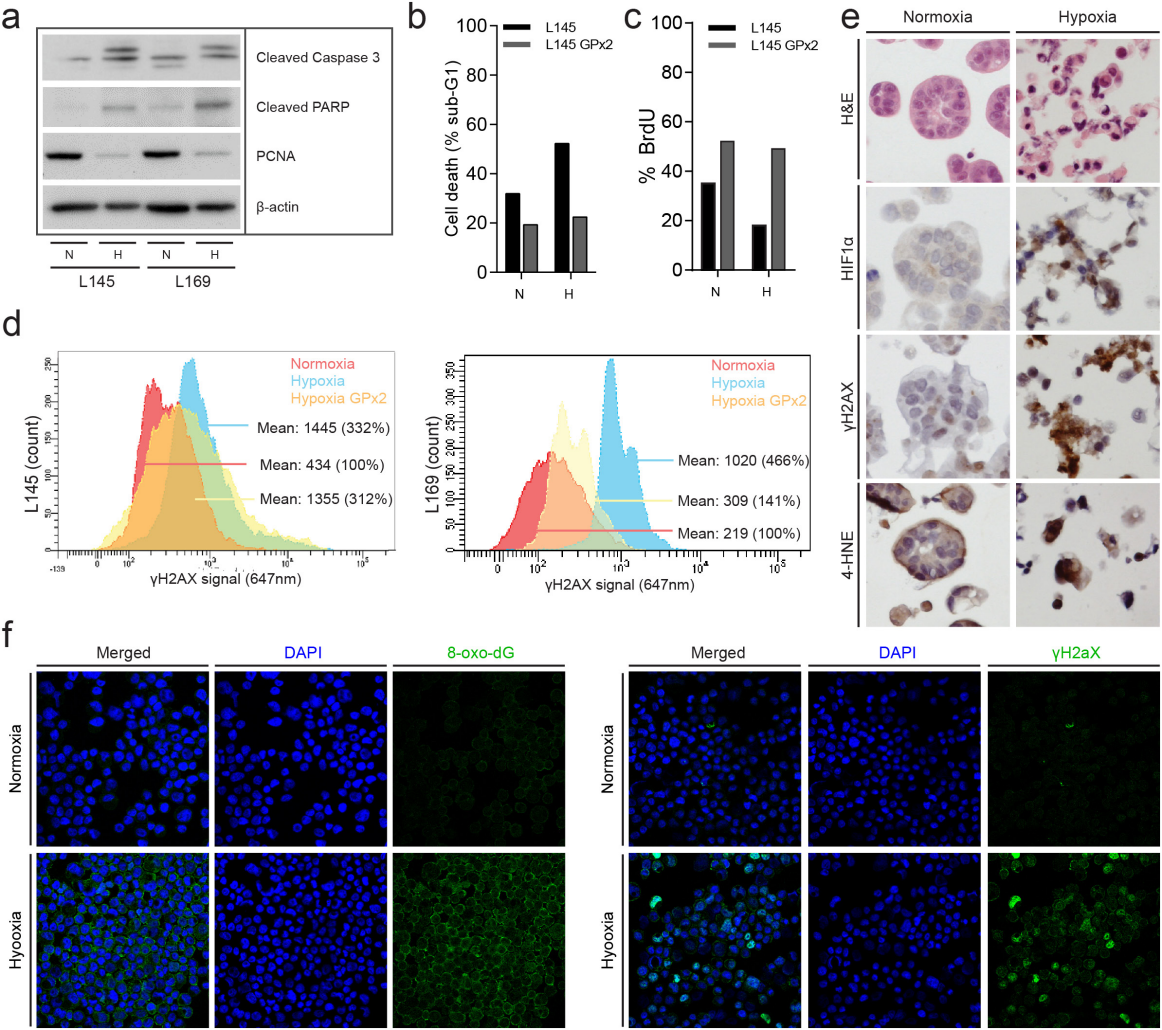
Chronic hypoxia induces oxidative damage and promotes tumor cell death **(a)** Human colonospheres were cultured in hypoxia (0.1%) and normoxia (21%) for 72 hours. Cells were lysed and analyzed by Western blotting for PCNA (proliferation) and cleaved caspase-3 (apoptosis) (cropped gels). **(b)** Experiment performed as in (a) but human colonospheres overexpressing GPx2 were included. FACS analysis of PI-stained cells was then used to assess cell death. The bar graph shows the percentage of cells with sub-G1 DNA content. **(c)** The culture conditions were similar to a and b. Cells were pulse labeled with BrdU just prior to FACS analysis to assess the percentage of proliferating cells. **(d)** FACS analysis of γH2AX levels in control and GPx2-overexpressing colonospheres. Cells were exposed to hypoxia for 24 hours. **(e)** Colonospheres were cultured in normoxia or hypoxia for 24 hours and were subsequently embedded in agar and fixed in formalin for IHC analysis of HIF1α, γH2AX and 4-HNE levels. **(f)** Colonospheres were cultured in normoxia or hypoxia for 24 hours. After single cell making living cells were FACS sorted and processed for immunofluorescence analysis of 8-oxo-dG (left panel) and γH2AX (right panel). DAPI was used to visualize cell nuclei.

Hypoxia leads to the generation of reactive oxygen species (ROS), which could contribute to DNA damage [27, 28]. To study the contribution of ROS generation to hypoxia-induced DNA damage we used colonospheres overexpressing glutathione peroxidase 2 (GPx2) [29]. GPx2 uses glutathione to neutralize peroxides and is one of the most powerful intracellular anti-oxidant enzymes. Overexpression of GPx2 reduces cellular H_2_O_2_ levels and protects cells against oxidative stress induced by chemotherapy, single cell making, or exogenous H_2_O_2_ [30]. We found that prolonged exposure to hypoxia (72 hours) reduced proliferation and increased caspase cleavage and cell death, presumably resulting from chronic DNA damage (Figure 5a). Hypoxia-induced effects on proliferation, caspase processing and cell death hardly occur in a cell line overexpressing GPx2. This indicates that the hypoxia mediated changes are at least in part due to generation of ROS (Figure 5b-5d). To further study hypoxia-induced oxidative damage we analyzed incorporation of the oxidized nucleotide 8-oxo-dG into DNA and accumulation of the oxidized lipid 4-hydroxy-nonenal (4-HNE). Immunohistochemistry analysis of 4-HNE on paraffin-embedded colonospheres showed an increased level of lipid peroxidation in colonospheres that had been exposed to hypoxia (Figure 5e). Likewise, immunofluorescence analysis of live FACS-sorted cells showed an accumulation of 8-oxo-dG in hypoxia-exposed colonospheres (Figure 5f). Accumulation is seen in both the nucleotide precursor pool and to a smaller extend in the nucleus [31]. Together the data show that prolonged hypoxia results in reduced proliferation and increased cell death which is accompanied by oxidative DNA and lipid damage.

### The hypoxia-activated pro-drug Tirapazamine (TPZ) reduces the stem-like Aldefluor^bright^ population *in vitro* and in liver metastases

**Figure 6:**
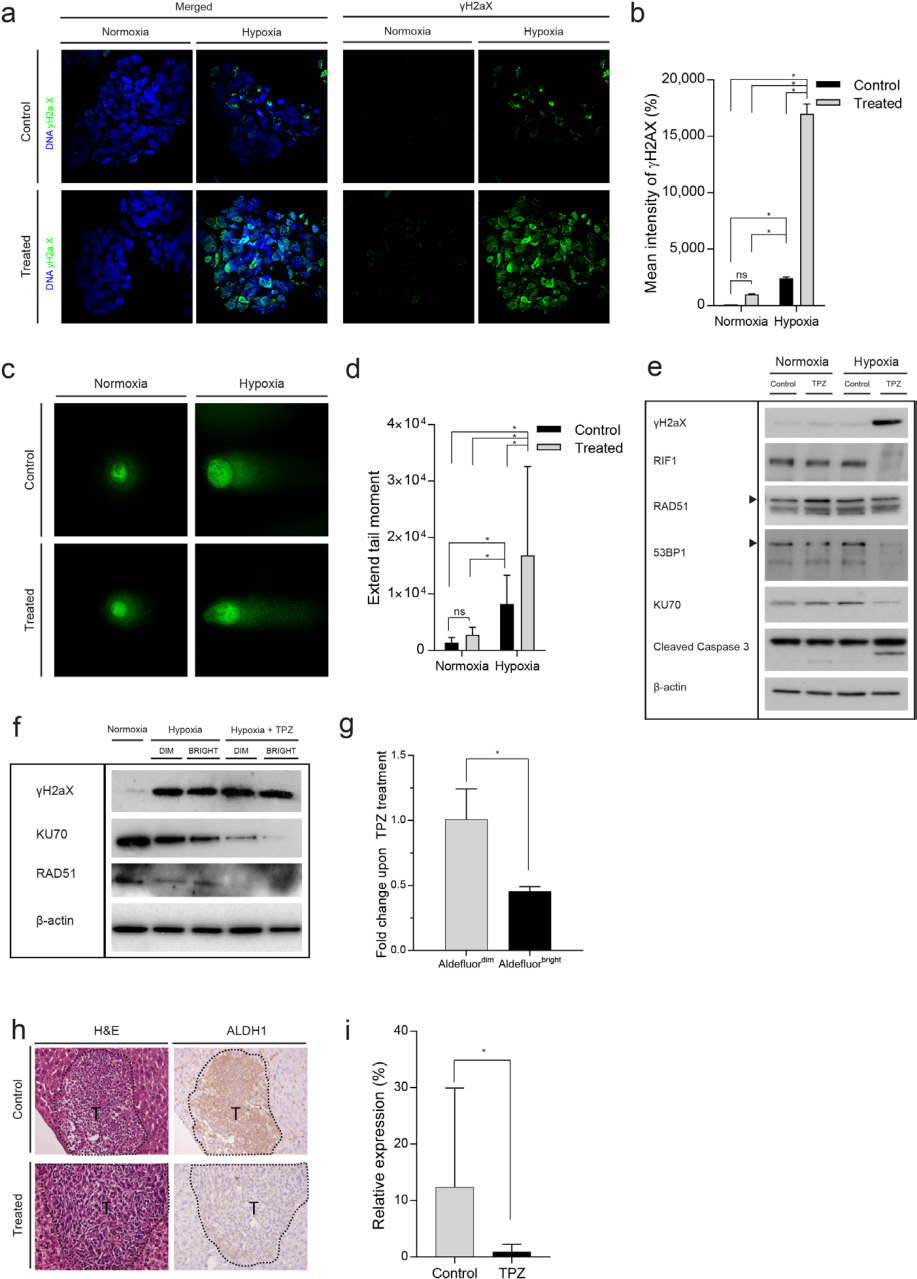
The hypoxia-activated pro-drug Tirapazamine (TPZ) reduces the stem-like Aldefluor^bright^ population *in vitro* and in liver metastases **(a)** Human colonospheres were cultured in hypoxia (0.1%) and normoxia (21%) for 24 hours in the absence or presence of TPZ for 4 hours. Cells were processed for immunofluorescence analysis of γH2AX (green). DAPI (blue) was used to visualize cell nuclei. **(b)** Bar graphs showing the quantification of γH2AX foci observed in (a), shown as percentage of the mean γH2AX intensity at normoxia (n=3). **(c)** Representative confocal pictures of the comet assay of colonospheres cultured as in (a). **(d)** Bar graphs showing the quantification of DNA damage observed with the comet assay (Extend tail moment) (n=77). **(e)** The experiment was performed as in (a), but cells were either treated or untreated for 16 hours. Cells were lysed and analyzed by Western blotting for the indicated markers (cropped). **(f)** Colonospheres were cultured in hypoxia and treated with TPZ for 16 hours. The cells were FACS sorted into Aldefluor^bright^ and Aldefluor^dim^ populations and analyzed by Western blotting for the indicated markers. **(g)** Human colonospheres were cultured in hypoxia (0.1%) for 24 hours in the absence or presence of TPZ for 4 hours and FACS sorted into Aldefluor^bright^ and Aldefluor^dim^ populations. The bar graph shows the fold change Aldefluor^dim^ and Aldefluor^bright^ cells upon TPZ treatment (n=3). **(h)** C26 tumor cells were injected into the liver parenchyma of immune-deficient mice. Following tumor initiation, the tumor-bearing liver lobes were subjected to a vascular clamping protocol [24] to induce hypoxia and treated with saline (control) or TPZ (treatment) for 10 days. At endpoint, the livers were excised and expression of ALDH1 was examined by IHC. Magnification 20x, T=tumor. **(i)** Bar graphs showing the quantification of ALDH1 (n=28) observed in (h) (n=14). * = significant (p>0.05). ns = not significant.

So far, the data indicate that DNA repair is reduced in hypoxic conditions, which may create a therapeutically exploitable vulnerability. Tirapazamine (TPZ) is a hypoxia-activated prodrug which causes primarily DNA double-strand breaks by inhibiting topoisomerase II [23]. To analyze DNA damage in TPZ-treated cells, we cultured human colonospheres in normoxia and hypoxia in the absence or presence of TPZ. Immunofluorescence (Figure 6a and 6b), comet assay (Figure 6c and 6d) and western blotting (Figure 6e) showed that TPZ strongly enhanced DNA double strand break formation (γH2AX) and apoptosis (as evidenced by caspase-3 cleavage) in hypoxic colonospheres. We also analyzed DNA repair protein expression in TPZ-treated cells. TPZ treatment in hypoxia caused strong suppression of RIF1, RAD51, 53BP1 and KU70 expression (Figure 6e). We previously showed that a subpopulation of stem-like cancer cells reside in hypoxic tumor niches [4, 6] from where they may drive tumor recurrence. Stem-like colon cancer cells can be identified by high aldehyde dehydrogenase (ALDH) activity, which is measured with the Aldefluor assay in which the Aldefluor^bright^ fraction contains the clonogenic and tumorigenic (i.e. stem-like) cancer cells [32–34]. Therefore, we tested whether TPZ would differently affect the induction of DNA damage and suppression of repair proteins in hypoxic Aldefluor^bright^ and Aldefluor^dim^ cells. Intact colonospheres were cultured in hypoxia and were treated with TPZ. After treatment, cells were FACS-sorted into Aldefluor^bright^ and Aldefluor^dim^ populations, and DNA damage and repair was analyzed in these populations by western blotting. DNA double strand break formation (γH2AX) was similar in Aldefluor^bright^ and Aldefluor^dim^ cells (Figure 6f). RAD51, and to a lesser extent KU70, were downregulated under hypoxia and expression of both repair proteins was further reduced by TPZ treatment. We found that TPZ reduced the Aldefluor^bright^ fraction in hypoxic colonospheres (Figure 6g and Supplementary Figure 4). The same results were found *in vivo*, as clamped liver lobes of mice treated with TPZ showed reduced levels of ALDH1 expression (Figure 6h and 6i).

## DISCUSSION

In the present study, we show that increased DNA damage, possibly caused by reduced repair protein expression, is a therapeutically exploitable feature of aggressive colon tumors. Besides, an increase in DNA damage can also be caused by hypoxia-induced increase of reactive oxygen species (ROS) levels, and ROS-induced damage to the nucleotide pool. The link between hypoxia and DNA damage was found in primary tumors, in resected liver metastases, and in residual tumor tissue following liver surgery. The latter finding is important as it suggests a potential avenue for the development of effective adjuvant therapy. Current adjuvant treatment regimens with standard chemotherapy have minimal effects on the survival of this patient population [35], HAPs may be employed to prevent outgrowth of residual tumor cells following an intentionally curative partial liver resection. Suppression of repair protein expression during hypoxia likely contributes to reduced DNA damage repair capacity, which can lead to accumulation of damage and tumor cell death without the addition of drugs. However, a hypoxia-activated double strand break-generating drug like TPZ greatly augments DNA damage generation in hypoxia and could be used to exploit the lower repair capacity that is intrinsic to the hypoxic state.

RNA analysis of the primary tumors showed a general inverse association between hypoxia and repair protein expression, regardless of the specific repair pathway. Although this may be partially explained by a general lower proliferation rate of hypoxic tumors [36], our data also shows that hypoxia has a rapid down-regulatory effect on the expression of some of the repair proteins, even preceding HIF stabilization and DNA damage induction. This is in line with previous studies [18, 20, 21] and demonstrates a direct link between hypoxia, reduced DNA repair, and the accumulation of DNA damage. Future work should reveal how hypoxia leads to decreased repair protein expression and to what extent this contributes to DNA damage accumulation. A more detailed insight into these mechanisms may result in the identification of key players in DNA damage control under hypoxia. In turn, this may lead to novel treatment strategies, based on the increased accumulation of DNA damage.

Regardless of the underlying mechanism, there was a striking enrichment of aggressive mesenchymal-type CMS4 tumors in the [HIF2α^high^/Repair-Protein^low^] quadrants, showing also decreased DFS in all cases examined. A potential explanation for the observed connection between low repair and decreased survival probability could be that lower repair capacity leads to an increased mutation rate and an increased chance of the generation of metastasis-competent (and/or therapy resistant) sub-clones. This suggests that hypoxia targeting, for instance with TPZ or related hypoxia-activated topoisomerase inhibitors like AQ4N [37] and Q6 [38], should be explored as a therapeutic strategy in selected patients with CMS4-type tumors [39, 40].

Interestingly, studies on DNA repair-deficient cancer syndromes like Lynch syndrome for CRC and BRCA-associated ovarian and breast cancer have led to the concept of synthetic lethality: the genetic deficiency of one repair pathway (e.g. mismatch repair) leads to a hypersensitivity to drugs targeting additional repair pathways (e.g. through PARP inhibition) [41, 42]. It will therefore be interesting to assess whether hypoxia - by suppressing the activity of one or more repair pathways - causes hypersensitivity to inhibitors of other repair pathways. Some studies indeed show that hypoxia sensitizes tumor tissue to PARP inhibitors in combination with radiation, supporting this treatment concept [19, 22, 43–46]. Although we have not found synergistic responses of hypoxic colon cancer cells and tumors to a combination of TPZ and the PARP inhibitor Olaparib (JJ, unpublished observations), this is an area that deserves further investigation. Novel platforms in translational oncology, including organoid culturing technology [47–49] and PDX-models [50] are now widely used to develop personalized cancer treatment. Interestingly, a subset of human colon tumors can only be established as organoids at low oxygen tension [49]. This hypoxia-addicted subgroup of colon cancer organoids can now be used in drug screens and transplantation models to identify treatment combinations with hypoxia-activated prodrugs and/or DNA repair inhibitors that effectively eradicate hypoxic tumor tissue.

Hypoxia within tumors is, by definition, heterogeneous. However, features of hypoxia are clearly overrepresented in the CMS4 subtype, providing a potential diagnostic strategy to select patients for hypoxia/repair-targeting therapy. Multiple robust diagnostic tools for selecting patients with CMS4 tumors have recently been developed and are now available [39, 40]. As an alternative to CMS4 tests, patients may also be selected by immunohistochemistry for hypoxia -, DNA damage -, and/or DNA DSB repair markers. In addition, hypoxia-generating procedures such as liver surgery or (radio-) embolization, may be followed by such therapy regardless of tumor subtype, aiming to kill hypoxic tumor residue and prevent recurrence. In such cases, hypoxia is externally generated rather than a consequence of tumor genetics. Another factor potentially influencing response to hypoxia/repair-targeting therapy is variation in DNA repair capacity between individuals and tumors. For instance, polymorphisms in miRNA binding sites in genes encoding DNA DSB repair genes can be used to study differences in DNA repair capacity in CRC patients [51]. Eventually, the analysis of such genetic variations may also have predictive value and could help select patients for hypoxia/repair targeting therapy.

Whatever the selection strategies will be, we propose that reduced repair capacity in a subset of human colorectal cancers and in post-treatment tumor tissue provides a clear opportunity for therapeutic intervention. DNA repair defects and sensitivity to hypoxia-activated topoisomerase inhibitors are the basis for further developing combination therapies aimed at eradicating hypoxic tumor tissue.

## MATERIALS AND METHODS

### Bioinformatics analyses

All bioinformatics analyses were performed by using the R2 Genomics analysis and visualization platform (http://r2.amc.nl). To visualize expression of particular gene sets in distinct tumor subgroups the option ‘relate two tracks’ was used. Condensation of gene set expression into single values per tumor was performed with the ‘View Gene Set’ option and storing the obtained values as a track for subsequent analysis. The HIF2α signature was previously published [4].

All dot plots visualizing the comparative expression of gene sets were created using the ‘relate two tracks option’. Pearson correlation^®^ values and accompanying p-values were obtained by selecting the xy plot option.

### Immunohistochemistry

Both human and mice tumors were immediately after respectively resection and necropsy fixed in 4-10% neutral-buffered formalin and embedded in paraffin. Spheroid pellets were fixed in 4% phosphate-buffered formalin, first embedded in a 2.5% agar droplet and subsequently embedded in paraffin. Serial sections (4μm) were used for immunohistochemical analyses and stained with CAIX (1:1000 ab15086, abcam), Hif1α (1:50, 610959, BD Transduction Laboratories), γH2AX (Ser139) (1:50, sc-2557, Cell signaling, Littleton, MA), RIF1 (1:16000, A300-569A, Bethyl), KU70 (A-9) (1:6000, sc-5309, Santa Cruz), RAD51 (1:100. HPA039310, Sigma Atlas), Anti-4 OH nonenal (1:100, ab46545, abcam), ALDH1 (1:500, 611195, BD Transduction Laboratories). Briefly, tissue sections were deparaffinized with xylene and rehydrated through a series ethanol concentrations. Antigen retrieval was achieved by cooking for 20 minutes with citrate or EDTA, followed by blocking of the endogenous peroxidase activity for 1 hour at room temperature with 0.3% H2O2. Primary antibody was either applied for 1 hour at room temperature (CAIX, RAD51, KU70) or overnight at 4 degrees of Celcius (γH2AX, HIF1α, 4-OH-HNE, RIF1). HRP-labelled secondary antibody (Powervision Immunologic, Immunovision Technologies, Brisbane, CA) was applied after washing and tissue sections were stained with DAB reagent (Dako). Nuclei were counterstained with Hematoxylin (Mayer). Tissue architecture and identification of necrotic areas was achieved by Hematoxylin and Eosin staining. Areas are subdivided into areas of necrosis (N), tumour (T), peri-necrotic (PN) and Reference Zone (RZ). The Novolink Polymer Detection System (Leica Biosystems Inc, Buffalo Grove, IL) was used according to the manufacture’s protocol for some stainings (HIF1α, γH2AX, 4HNE). Staining was analyzed by ImageJ after subtracting background staining. Areas were defined as microscopic fields (20x).

### Patient-derived colonosphere culture

Human colonosphere cell lines (L145 and L169) were derived from patients harboring colorectal liver metastases and cultured in stem cell medium as described previously [14]. Generation of colonospheres stably expressing GPx2-expression constructs was established as described before [29]. All cell culture was carried out at 37°C in a 5% CO_2_-humidified incubator under normoxic (21% O_2_) or hypoxic (0.1% O_2_) conditions, the latter using an *In vivo* 2 Hypoxia Workstation (Biotrace International, Spennymoor, UK). Tumor tissue was obtained in accordance with the local medical ethical committee on human experimentation (protocol #09-145). Informed consent was obtained from all patients. Colonospheres were cultured with or without Tirapazamine (TPZ) (20 μM; kindly provided by Dr Minchinton, BC Cancer Agency, Vancouver, Canada) for the indicated time.

### Immunofluorescence

Colonospheres were harvested and fixed for 20 minutes in PBS containing 4% of formaldehyde and permeabilized with 1% Triton X-100 at room temperature for 10 minutes and overnight in ice-cold (−20°C) methanol or PBS. Cells were blocked in PBS containing 0.1% Tween and 5% BSA and incubated overnight at 4°C with primary antibodies in PBS containing 0.1% Tween and 2% BSA; p-γH2AX (Ser139) (1:100, 05-636, Millipore), Anti-8 OH guanosine (1:200 ab48508, abcam). Colonospheres were subsequently washed and incubated for 1 hour at room temperature with secondary antibody (goat anti-mouse Alexa Fluor568; Invitrogen) in PBS containing 0.1% Tween and 2% BSA. DAPI (0.5 μg/mL) was used to stain the nuclei. Images (Z-stacks) were acquired using a Zeiss LSM510 Meta Confocal microscope. All images were acquired with identical illumination settings and analyzed in 3D with Imaris version 8.2 Software (Bitplane AG, Zurich, Switzerland).

### Comet assay

The OxiSelect Comet Assay was used to analyze cellular DNA damage using single cell electrophoresis (Cell Biolabs, San Diego, CA, USA) according manufacturer instructions. In short, cells were trypsinized, washed and diluted to 10^5^/ml in PBS. Cells were then added to low-melting temperature agarose (10^4^/ml final) and immediately plated (75 μl) on comet assay glass slides (Cell Biolabs) coated with normal-melting temperature agarose. After lysis, slides were placed in a horizontal gel electrophoresis chamber and covered with an alkaline buffer (5 mM NaOH and 200 mM Na_2_EDTA; pH >13). Following a 30 minute DNA “unwinding” period, electrophoresis was performed under standard conditions (21 V, 300 mA; distance between electrodes = 20 cm) for 30 minutes. Following neutralization to pH 7.5 using Trizma base (Sigma-Aldrich, St Louis, MO, USA), gels were stained with Vista Green DNA dye and stored at 4°C until analysis. Images were acquired with a Zeiss LSM 510 confocal microscope and LSM 710 version 3.2SP2 software. DNA damage was quantified per the manufacturer's instructions by calculating the extent tail moment: Extent Tail Moment = Tail DNA% × Length of Tail; where Tail DNA% = 100 × Tail DNA Intensity/Cell DNA intensity. For each time point, means ± standard error of the mean were calculated. Statistical analysis was performed using an unpaired Student's t-test.

### Western blot analysis

Lysates of colonospheres were prepared using laemmli buffer. Equal amounts of protein were loaded and run out on sodium dodecyl sulphate-containing gels and blotted onto nitro-cellulose membranes. Western blotting was performed using standard protocols. The following antibodies were used: Hif1α (1:500, 610959, BD Transduction Laboratories), Hif2α (1:500, ab8365, Abcam, Cambridge, MA), Cleaved Caspase 3 (Asp175) (1:1000, 9661, Cell Signaling, Littleton, MA), anti-PCNA (1:1000, SC-56 Santa Cruz), 53BP1 (1:1000, nb100-304 Novus Biologicals, Littleton, CO), p-γH2AX (Ser139) (1:1000, 05-636, Millipore), RIF1 (1:2000, A300-569A, Bethyl,), KU70 (A-9) (1:200, sc-5309, Santa Cruz,), RAD51 (1:2500, ABE257, Millipore), cleaved PARP (1:1000, 9541, Cell Singalling, Littleton, MA), LC3 (5F10)(1:1000, 0231-100, Nanotools antibodies, Teningen, Germany) β-Actin AC15 (1:20000, NB600-501, Novus Biologicals, Littleton, CO).

### Proliferation assay

A DNA synthesis–based cell proliferation assay using 10 mM 5-Bromo-2´-Deoxyuridine (BrdU) (BD Biosciences, Franklin Lakes, NJ, USA) was performed according manufacturer instructions.

### Flow cytometry

L145 and L169 (with or without GPx2 construct) colonosphere cell lines were cultured under normoxia or hypoxia for the indicated time points. FACS analyses for γH2AX is performed according to the previous published protocol [52] in combination with 7-AAD for cell viability. We used γH2A.X (Ser139) (1:200, sc-2557, Cell signaling, Littleton, MA), via-probe cell viability solution (7-AAD) (10μl/1×10^6^ cells, 555816, BD Transduction Laboratories), RNAse 10mg/ml 1:400. Aldefluor activity and γH2AX were analyzed by fluorescence-activated cell sorting (FACS) using DIVA software (BD Biosciences). For the Aldefluor experiments, colonosphere line L145 was either first treated or first sorted. If first treated, cells were cultured for the indicated time under normoxic or hypoxic conditions with or without TPZ (20μM). The concentration of TPZ (20μM) was based on previous work in our lab [6]. Next, cells were trypsinized to obtain single-cell suspensions. Cell doublets and clumps were excluded by prior filtration with a 40μM filter and by using doublet discrimination gating during FACS analysis. Nonviable cells were excluded based on 7-AAD expression. Aldefluor positive cells were analyzed according to the manufacturer’s protocol by using the ALDH (aldehyde dehydrogenase isoform 1) substrate BAAA (1μmol/L per 1×106 cells; StemCell Technologies, Vancouver, Canada). Negative control samples were co-incubated with diethylaminobenzaldehyde (50 mM, StemCell Technologies). When indicated, cells were first sorted based on Aldefluor positivity and afterwards cultured for the indicated time under normoxic or hypoxic conditions with or without TPZ (20μM). Afterwards, percentage of γH2AX expression in L145 cells was determined by immunofluorescence or Western blot as described. The cell sorting experiments were conducted with a 6-color FACS Aria III Cell Sorter (Becton Dickinson Biosciences, Mountain View, CA).

### Animals

All experiments were performed in accordance with the guidelines of the Animal Welfare Committee of the University Medical Center Utrecht, The Netherlands. Male Balb/C mice (10-12 weeks) were purchased from Charles River (Sulzfeld, Germany) and were housed under standard laboratory conditions.

### Murine model of hepatic hypoxia

A mouse model for hepatic hypoxia was used as described previously [24], in which hypoxia was induced through vascular clamping of the left liver lobe for 45 minutes. TPZ was then administered one day after clamping for a period of 10 days. The livers were excised, formalin fixed and paraffin embedded and expression of ALDH1 was examined by IHC.

### Statistical analyses

Box and Whisker plots are shown to display the variation of CMS in the DNA repair patterns. Scatter plots are used for regression analysis and shown by^®^. Data is presented as mean ± SD. The Student t test (unpaired, two-tailed) was performed to analyze if differences between the groups are statistically significant, using SPSS version 23 (IBM SPSS Statistics, Armond, NY) and GraphPad Prism version 5.0 (Graphpad Software, La Jolla, CA). Differences with a P value of less than 0.05 were considered statistically significant. Box and whisker plots showed non-normal distribution of the used parameters (data not shown), therefore correlation is analyzed by Spearman’s rank correlation coefficient correlation.

## SUPPLEMENTARY MATERIALS FIGURES AND TABLE





## Abbreviations

4-HNE: 4-hydroxy-nonenal
ALDH: aldehyde dehydrogenase
CAD: caspase-activated DNAse
CMS4: consensus molecular subtype 4
CSC: cancer stem cell
FACS: fluorescence-activated cell sorting
GPx2: glutathione peroxidase 2
HAPs: hypoxia-activated prodrugs
HIF1α: hypoxia-inducible factor 1 alpha
HIF2α: hypoxia-inducible factor 2 alpha
HR: homologous recombination
NHEJ: non-homologous end-joining
ROS: reactive oxygen species
TPZ: Tirapazamine
γH2ax: phosphorylated histone 2AX
