# Supplementary material for: Downregulation of DNA repair proteins and increased DNA damage in hypoxic colon cancer cells is a therapeutically exploitable vulnerability
Source: Oncotarget. 2017 Sep 21;8(49):86296–311. doi: 10.18632/oncotarget.21145 (PMC5689686; doi:10.18632/oncotarget.21145)
Supplement: Supplementary file 1 [file oncotarget-08-86296-s001.pdf]

Downregulation of DNA repair proteins and increased DNA damage in hypoxic colon cancer cells is a therapeutically exploitable vulnerability

SUPPLEMENTARY MATERIALS

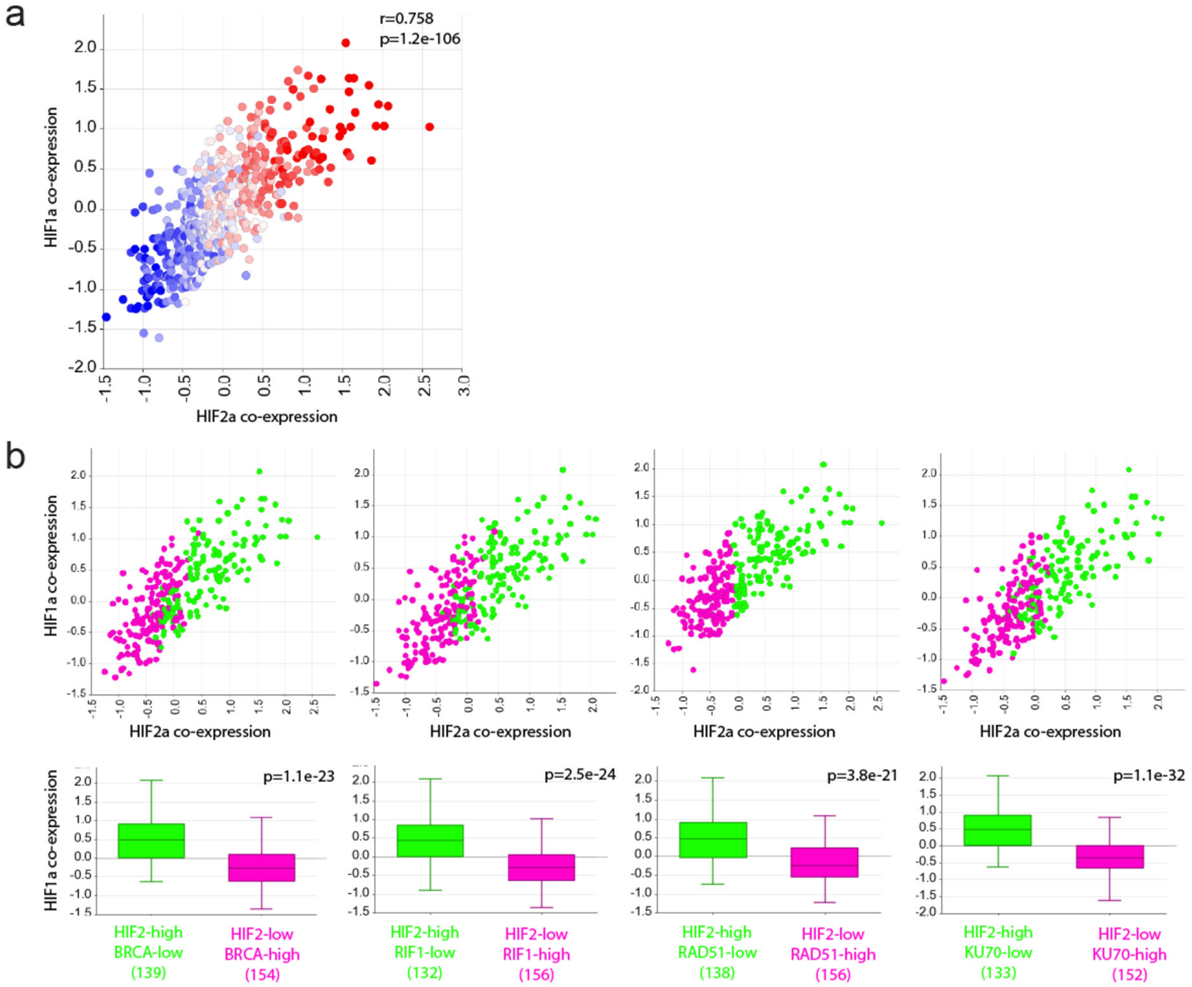

Supplementary Figure 1: Co-expression of HIF1a and HIF2a signatures shows an inverse correlation with repair gene expression. (a) Expression of HIF1alpha and HIF2alpha co-expression signatures was determined in 566 tumors of the CIT566 cohort. XY plot analysis shows highly significant co-expression of both signatures. Expression of the signature identifying mesenchymal-type tumors (CMS4) is color-coded from blue (low) to red (high). **(b)** Expression of the HIF1a signature is significantly higher in all HIF2a-HIGH/repair protein-LOW tumor subgroups. Tumors are color-coded as HIFlow (pink) and HIFhigh (green).

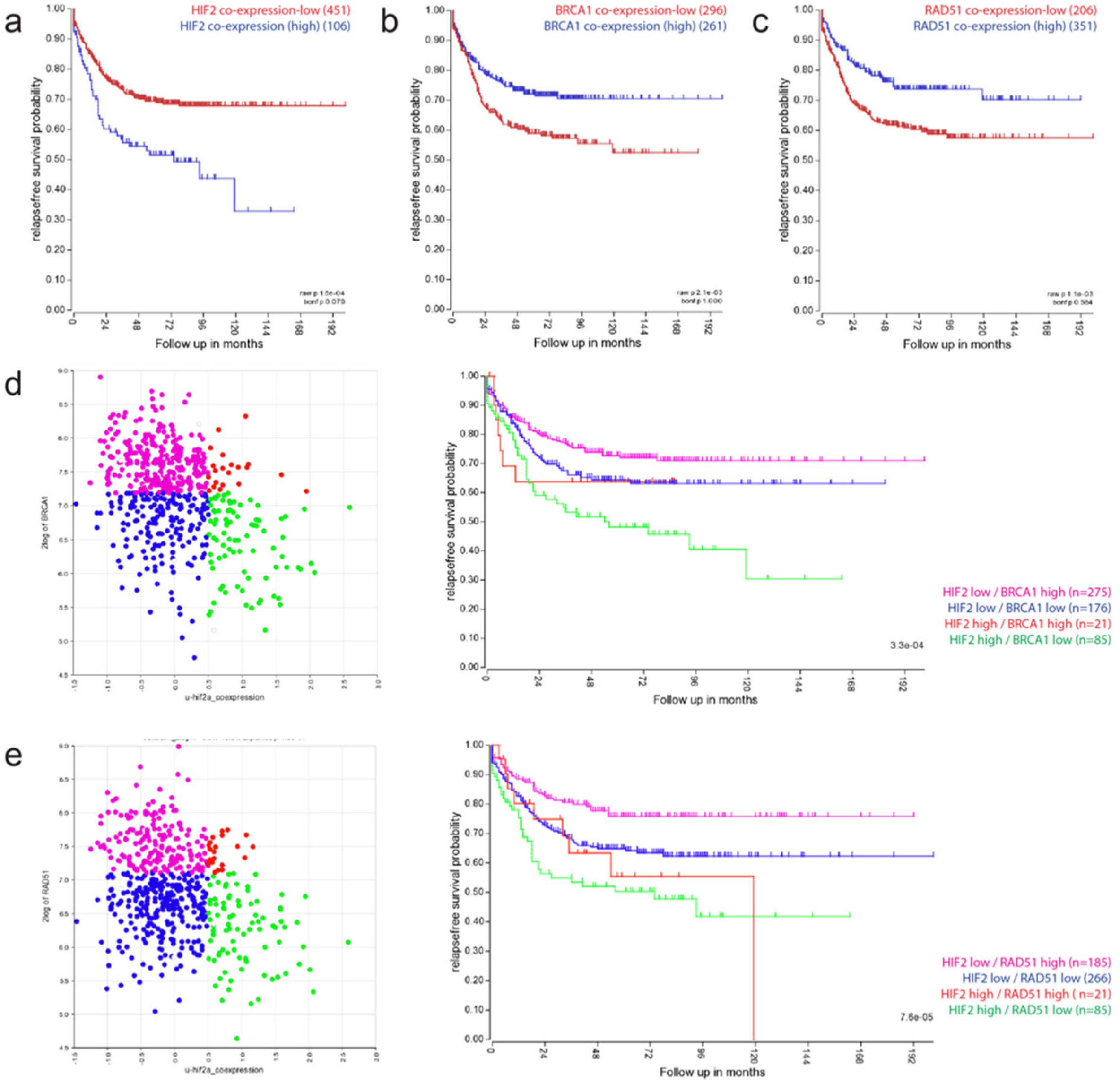

Supplementary Figure 2: HIF2-high/repair-low tumors have a poor prognosis. (a-c) Kaplan scan was performed in R2 (http://r2.amc.nl) to identify the optimal cutoff points for the HIF2 co-expression signature, BRCA1 and RAD51. The optimal cutoff points are indicated by the tumor numbers in each group. These cutoff points were then used to generate new quartiles, as in Figure 1c. **(d-e)** Kaplan Meier curves showing the survival differences between the four HIF2-BRCA and HIF2-Rad51 subgroups, based on the Kaplan-scan generated cutoff points.

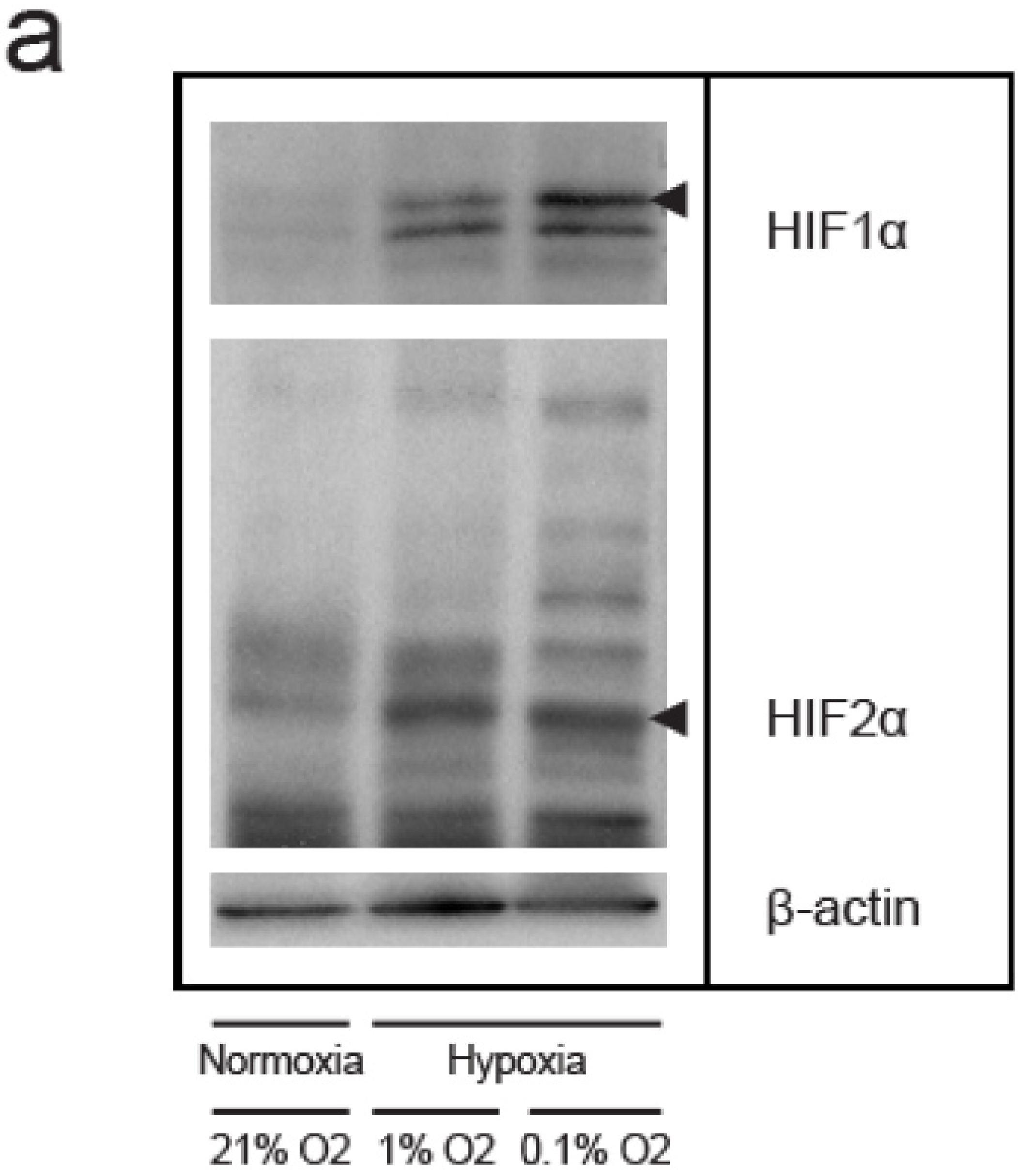

Supplementary Figure 3: In vitro exposure to hypoxia upregulates both HIF1α and HIF2α. (a) Human colonospheres were cultured in normoxia (21% O_2), hypoxia (1% O_2) or hypoxia (0.1% O_2) for 24 hours. Cells were lysed and analyzed by Western blotting for the hypoxia markers Hif1αand Hif2α.

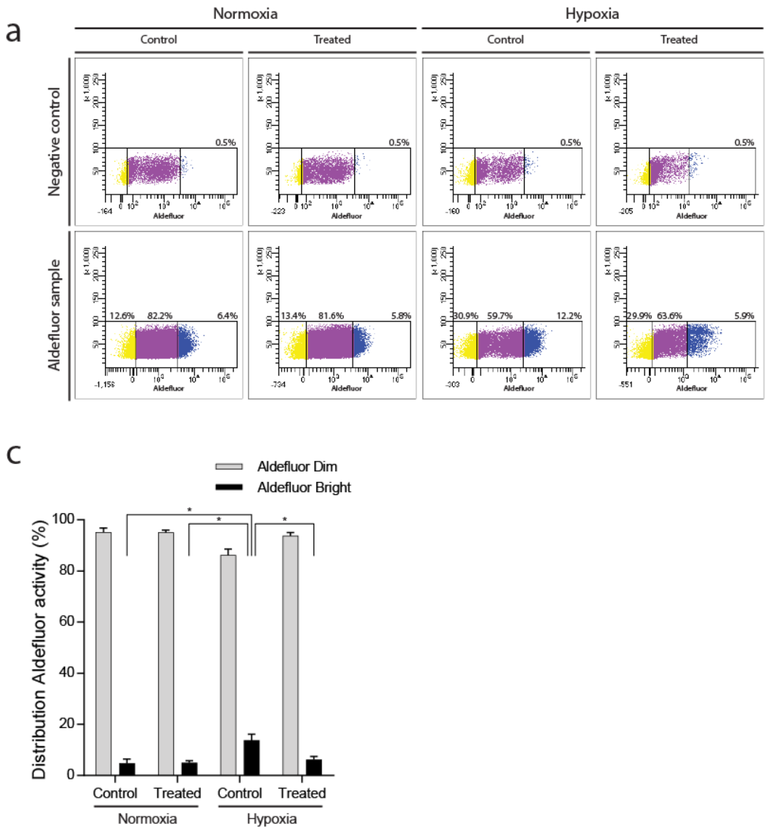

b

	Normoxia		Hypoxia	
	Control	Treated	Control	Treated
AldefluorNegative	12,6%	13,4,8%	30,9%	29,9%
AldefluorDIM	82,2%	81,6%	59,7%	63,6%
AldefluorBRIGHT	6,4%	5,8%	12,2%	5,9%

	Normoxia		Hypoxia	
	Control	Treated	Control	Treated
AldefluorNegative	23,7%	22,4%	28,0%	28,7%
AldefluorDIM	98,1%	74,7%	66,8%	68,2%
AldefluorBRIGHT	1,7%	2,9%	5,2%	3,1%

Supplementary Figure 4: Hypoxia induced increase of CSC is abolished by TPZ treatment. (a) Colonospheres cultured in normoxia or hypoxia, untreated or treated with TPZ and FACS-sorted based on Aldefluor activity. Yellow = Aldefluor^{Negative}, Purple = Aldefluor^{dim}, Blue = Aldefluor^{bright}. **(b)** Hypoxia- and TPZ-induced changes (in percentage) of Aldefluor activity in colonospheres. **(c)** Human colonospheres were cultured in hypoxia (0.1%) and normoxia (21%) for 24 hours in the absence or presence of TPZ for 4 hours. Cells were FACS sorted into Aldefluor^{bright} and Aldefluor^{dim} populations. Bar graphs showing the distribution of Aldefluordim/Aldefluorbright cells (n=3). * = significant (p>0.05).

Supplementary Table 1: Correlation of the HIF2a co-expression signature with DNA repair genes

See Supplementary File 1
